# Strain-Insensitive Hierarchically Structured Stretchable Microstrip Antennas for Robust Wireless Communication

**DOI:** 10.1007/s40820-021-00631-5

**Published:** 2021-04-09

**Authors:** Jia Zhu, Senhao Zhang, Ning Yi, Chaoyun Song, Donghai Qiu, Zhihui Hu, Bowen Li, Chenghao Xing, Hongbo Yang, Qing Wang, Huanyu Cheng

**Affiliations:** 1 Department of Engineering Science and Mechanics, The Pennsylvania State University, University Park, PA 16802 USA; 2 Division of Life Sciences and Medicine, School of Biomedical Engineering (Suzhou), University of Science and Technology of China, Hefei, 230022 People’s Republic of China; 3 Institute of Biomedical Engineering and Technology, Chinese Academy of Science, Suzhou, 215011 People’s Republic of China; 4 Department of Materials Science and Engineering, The Pennsylvania State University, University Park, PA 16802 USA; 5 School of Engineering and Physical Sciences, Heriot-Watt University, University Park, Edinburgh, EH14 4AS Scotland, UK; 6 Department of Electrical Engineering, The Pennsylvania State University, University Park, PA 16802 USA

**Keywords:** Stretchable microstrip antennas, Strain-insensitive resonance frequency, Wireless communication, RF energy harvesting, Wearable and bio-integrated electronics

## Abstract

As the key component of wireless data transmission and powering, stretchable antennas play an indispensable role in flexible/stretchable electronics. However, they often suffer from frequency detuning upon mechanical deformations; thus, their applications are limited to wireless sensing with wireless transmission capabilities remaining elusive. Here, a hierarchically structured stretchable microstrip antenna with meshed patterns arranged in an arched shape showcases tunable resonance frequency upon deformations with improved overall stretchability. The almost unchanged resonance frequency during deformations enables robust on-body wireless communication and RF energy harvesting, whereas the rapid changing resonance frequency with deformations allows for wireless sensing. The proposed stretchable microstrip antenna was demonstrated to communicate wirelessly with a transmitter (input power of − 3 dBm) efficiently (i.e., the receiving power higher than − 100 dBm over a distance of 100 m) on human bodies even upon 25% stretching. The flexibility in structural engineering combined with the coupled mechanical–electromagnetic simulations, provides a versatile engineering toolkit to design stretchable microstrip antennas and other potential wireless devices for stretchable electronics.

**Supplementary Information:**

The online version contains supplementary material available at (10.1007/s40820-021-00631-5).

## Highlights

- The “ordered-unraveling” of hierarchical structures from mechanical assembly contributes to not only the improved overall stretchability, but also the small resonance shift of the stretchable microstrip antenna upon stretching.
- The mechanical-electromagnetic properties of stretchable microstrip antennas can be controlled by changing the number of arches in the meshed patch.
- A double-arched microstrip antenna was demonstrated to communicate wirelessly upon 25% stretching or when being placed on human bodies.

**Supplementary Information:**

The online version contains supplementary material available at (10.1007/s40820-021-00631-5).

## Graphical abstract

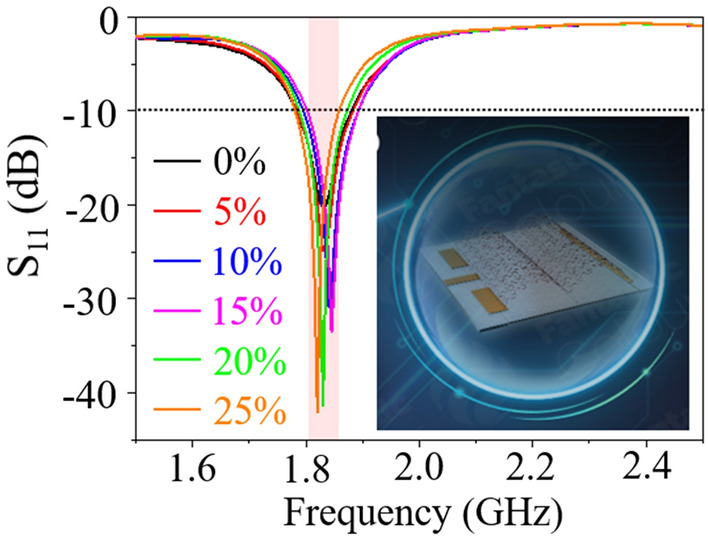

## Introduction

Flexible/stretchable electronics attached to dynamically changing, curvilinear surfaces can still function properly upon various mechanical deformations, including stretching, bending, and twisting [1]. The integration of sensors and actuators with data communication and powering modules in this class of emerging electronics enables its applications in energy generator/storage [2–6], human–machine interfaces [7, 8], health monitoring [9, 10], and clinical treatments [11]. The commonly used strategies in the design and fabrication of flexible/stretchable electronics rely on either intrinsically stretchable materials [12, 13] or stretchable structures [14–18]. The former includes the intrinsically stretchable semiconductors, insulators, and conductors. With direct relevance to wireless devices such as antennas, intrinsically stretchable conductors often explore conductive polymers [19], liquid metals [20], and conductive composites with low-dimensional nanofillers in a stretchable polymeric matrix [21]. Alternatively, the latter stretchable structures (e.g., serpentine [22–24] or 3D structures [14–16, 25]) allow the conventional metals to be stretched over tens of percent without damage. Compared to the intrinsically stretchable materials with various functionalities, conventional metal conductors (and semiconductors) with stretchable structures exhibit high performance comparable with modern electronics. Additionally, they can easily integrate the other commercial off-the-shelf (COTS) chips through a conventional soldering process to achieve extended capabilities for mass-production and commercialization.

As an indispensable component in flexible/stretchable electronics, wireless technology allows wireless powering [24] and data transmission [26–28] in the resulting miniaturized, integrated systems. For instance, near-field communication (NFC) has been widely used in wireless epidermal electronic systems [26] to monitor various physiologically relevant signals. However, its applications are limited by the short working distance. On the other hand, far-field communication with a radiofrequency (RF) antenna can be leveraged for wireless data and energy communication at a much longer working distance [10]. The combination of a RF antenna and rectifying circuit results in a rectifying antenna (rectenna) to harvest ambient RF energies for low-power flexible/stretchable electronics [29–31]. As a result, the development of stretchable antennas and rectennas started to gain momentum recently. Compared to the dipole and loop antennas, the microstrip patch antennas showcase significantly improved on-body performance because the ground plane can help eliminate or reduce the effect from the underlying lossy tissues [32]. In contrast to stretchable antennas based on liquid metals [33–35], conductive textile [36], or conductive composites [37], the stretchable metal antennas exhibit enhanced radiation efficiency due to the low ohm loss.

The resonance frequency of stretchable antennas with either stretchable materials or structures, shifts with tensile deformations, i.e., the frequency detuning [32, 33, 38, 39]. As a result, the stretchable antennas are only applied as wireless sensors based on the frequency shift [33, 39]. It is highly desirable to design stretchable antennas with strain-insensitive resonance frequency for robust wireless communication and RF energy harvesting. Although a stretchable monopole antenna with a relatively wide band has been demonstrated for communication, its radiation performance degrades when used the on-body [40] because of the absence of a ground plane. Here, we report hierarchically structured stretchable microstrip antennas with a meshed patch mechanically popped-up into a 3D shape to showcase the strain-insensitive resonance frequency over a large range of stretching up to 25%. The strain-insensitive resonance frequency comes from the cancellation of two effects, i.e., the increasing (or decreasing) resonance frequency of stretchable microstrip antennas with a meshed (or arched) patch as stretching increases, as revealed in our prior report [18]. The ground plane in the antenna also minimizes the effect from lossy human tissues when the antenna is used the on-body [41, 42]. The resulting stretchable microstrip antenna with a strain-insensitive resonance demonstrates excellent on-body performance from wireless communication to RF energy harvesting.

## Experimental Section

### Measurement of Dielectric Properties of Elastomeric Substrates

The elastomeric Ecoflex substrates were fabricated by mixing parts A and B with a ratio of 1:1. After curing at room temperature for 3 h, the Ecoflex samples in a square shape of 15 by 15 mm with a thickness of 1 mm were prepared for dielectric property measurements. A resonant mode dielectrometer (RMD-C-100, GDK Product Inc.) was used to measure the dielectric constant and loss tangent over the frequency range from 1 to 10 GHz. Input microwaves from a vector network were used to interact with the cavity of the resonant mode dielectrometer. The shift in resonance mode and the corresponding quality factor between the measurements with and without the sample determined the dielectric constant and loss of the sample. The measurements indicated that the dielectric properties underwent a negligible change in the frequency range from 1 to 5 GHz. Therefore, the dielectric constant of 3.125 and loss tangent of 0.01 (at 2.4 GHz) were used for the Ecoflex substrate in the design of stretchable antennas. The good agreement between the simulated and measured resonance frequency verified the accurate measurement of dielectric properties.

### Fabrication of the Stretchable Microstrip Antennas

The meshed layout designed in AutoCAD software was imported into the control system of an ultraviolet picosecond laser system (BX15, Edgewave). Commercial copper foils with a thickness of 20 μm (BangKai) fixed on a silicon wafer temporarily by the capillary force of water were patterned into the programmed mesh design by the laser system. The cutting power and speed of 0.4 μJ and 2000 mm s^−1^ were optimized to achieve the best spatial resolution. A soft adhesive silicone gel (v1510, Valigoo) was used to assemble the patterned patch and ground onto the Ecoflex substrate in a rectangular shape of 50 × 50 mm^2^ with a thickness of 1.5 mm. Depending on the number of arches (i.e., single- or double-arched), the meshed patch and ground were selectively bonded to the prestrained Ecoflex substrate with a pre-strain of 5%, 10%, or 15%. The release of the pre-strain resulted in the arched microstrip antennas with arched patch and ground. When the selectively bonded ground was replaced by a fully bonded ground, the stretchable antenna with an arched patch and a meshed ground was obtained. The meshed microstrip antenna was obtained by the full bonding of the meshed serpentine patch and ground to the Ecoflex substrate without the pre-strain. Soldering the as-fabricated antennas with a SubMiniature version A (SMA) connector using a soldering iron (Sn42Bi58, KZ-1513) completed the fabrication process.

### Measurement of Radiation Properties of Microstrip Antennas

The tensile strain in the range from 0 to 25% was applied to the stretchable microstrip antenna by a custom-built stretcher. Cylinders with different radii were used in the bending test of the antenna. The reflection curves (*S*_11_) of the deformed microstrip antenna were measured by a vector network analyzer (T5260A, Transcom Instruments). The radiation patterns were measured in an anechoic chamber. The on-body performance of the antenna was measured by attaching it to the arm of a healthy human subject with Magic tape.

### Wireless Communication of the Stretchable Microstrip Antenna

The commercial RF evaluation kit (SmartRF06) was employed to measure the wireless communication performance of the stretchable microstrip antenna. Two boards integrated with the CC2538 RF chip act as the transmitter and receiver, respectively. The chip can be programmed to transmit the RF energy at a power of − 3 dBm (0.50 mW). A PCB-based monopolar antenna was connected with the transmitter to provide omnidirectional radiation. The double-arched stretchable microstrip antenna resonating at 2.45 GHz was connected with the receiver to wirelessly communicate with the transmitter. The receiver was programmed to have a sensitivity of − 100 dBm. The receiving power at different distances for the stretchable microstrip antenna placed in the free space or on the human skin with stretching was measured, respectively.

## Results and Discussion

**Fig. 1:**
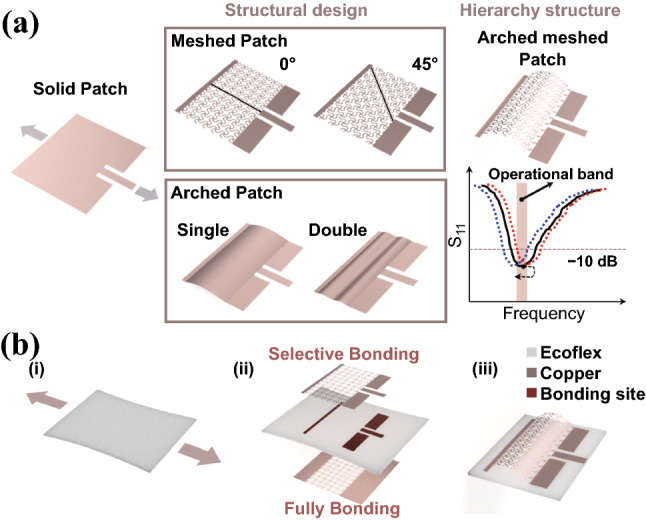
Schematic to demonstrate the design strategies of hierarchically structured stretchable microstrip antennas with horseshoe unit cells in a meshed layout and a 3D arched structure. **a** Design parameters in the stretchable structures include the orientation of the meshed layout and the number of 3D arches. In addition to an improved stretchability, the programmable mechanical-electromagnetic properties in the hierarchically structured stretchable microstrip antennas allow them to be strain-sensitive or strain-insensitive, with the former for wireless sensing and the latter for wireless communication and RF energy harvesting. **b** Fabrication process of the hierarchically structured antenna from the mechanical assembly. (**i**) After the soft Ecoflex substrate is pre-stretched, (**ii**) the meshed Cu structure patterned by laser is attached to the pre-stretched substrate with selective bonding sites, (**iii**) release of the pre-strain lifts the non-bonded region to form a 3D structure

Stretchable structures such as horseshoe unit cells and arched elements have been explored to result in stretchable microstrip antennas with conventional metals. The combination of the two design strategies into the hierarchically structured microstrip antennas (Fig. 1a) provides improved stretchability and versatile tuning of mechanical-electromagnetic properties because of the programmed structure unfolding upon stretching. Mechanical assembly of the meshed metal patch and ground with horseshoe unit cells on a soft Ecoflex substrate (dielectric constant and loss tangent of 3.125 and 0.01) results in the stretchable microstrip antennas with hierarchical structures (Fig. 1b). Although the horseshoe unit cells with different characteristic dimensions can be arranged in a periodic lattice (e.g., rectangular, triangular, or hexagonal) [23], this study simply uses the anisotropic square lattice structure with different mesh orientations to demonstrate the design concept. In brief, the fabrication starts with the laser patterning of the meshed metal patch and ground with horseshoe unit cells. Next, the meshed metal patch and ground are selectively bonded to a pre-stretched Ecoflex substrate. The release of the pre-strain mechanically lifts the regions that are not bonded to the substrate to form 3D structures because of the compressive forces (Fig. 1b). When fully bonded, the deformation of the meshed lattice structures in the out-of-plane direction is minimized. The full bonding of wavy meshed ground is applied for easy integration of the antenna on various curvilinear surfaces in the following studies unless otherwise specified. An inset microstrip line with a characteristic impedance of 50 Ω is then used to feed the stretchable microstrip patch antenna. Compared to the probe feeding [32], this in-plane feeding provides easy integration with other COTS chips and electrical components. Although the mechanical properties (e.g., stress–strain curve and stretchability) of the horseshoe unit cells and resulting lattice structures have been shown to depend on the characteristic dimensions, the effect of the orientation of the square lattice with respect to the feeding direction on the mechanical and radiation properties is yet to be investigated. After revealing the orientation effect, the stretchable antennas with mechanically assembled lattice structures are then reported. The tunable mechanical-electromagnetic properties allow the stretchable microstrip antenna to be designed with strain-sensitive or strain-insensitive properties, with the former for wireless strain sensing and the latter for wireless on-body communication.

### Meshed Microstrip Antennas for Wireless Strain Sensing

**Fig. 2:**
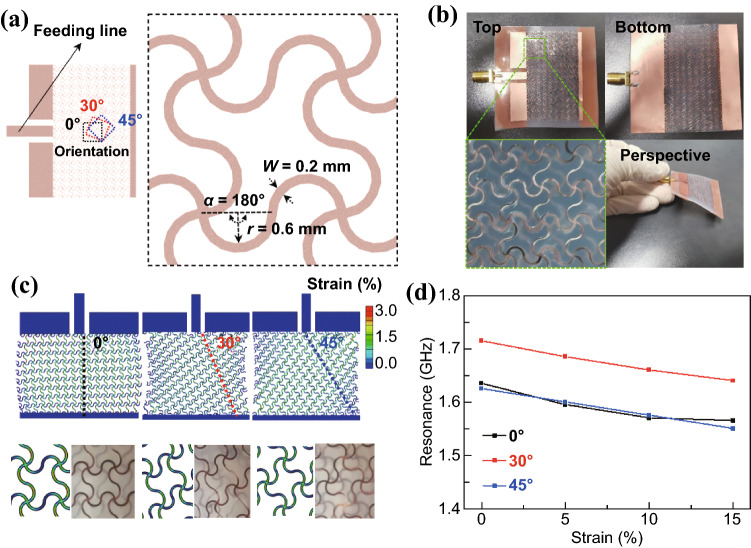
Mechanical-electromagnetic properties of the meshed microstrip antenna. **a** Schematic to show the meshed patch composed of horseshoe unit cells in a meshed layout with three different orientations (0˚, 30˚, and 45˚) relative to the feeding line. **b** Optical images of the meshed microstrip antenna with the 0˚ orientation. **c** Strain distributions in the meshed patch with different orientations upon 15% stretching. Optical images of the meshed patch upon a tensile strain of 15% from the experiment are included for comparison. **d** Measured resonance frequency of the meshed microstrip antennas with three different orientations as a function of the applied tensile strain

Because the square has rotational symmetry of order 4, this work considers three representative orientations (i.e., 0°, 30°, and 45°) (Fig. 2a). The geometric parameters of horseshoe unit cells remain unchanged in the study of orientation effects (i.e., line width *w* = 0.2 mm, arc radius *R* = 0.6 mm, and arc angle *α* = 180°). The microstrip antenna with a solid patch and ground is designed to resonate at 2.4 GHz. Replacing the solid patch and ground with horseshoe square lattice structures (i.e., the stretchable meshed microstrip antenna in Fig. 2b) lead to the shift of the resonance frequency to a lower value (Fig. S1), which is consistent with the previous report [18, 43]. Though the apparent physical dimensions of the microstrip antennas with horseshoe square lattice structures are not changed from their solid counterparts, the horseshoe lattice structures increase the equivalent wavelength in the current path. According to the cavity model [44], the increased effective dimension of the cavity from the increased current path results in a reduced resonance frequency. The meshed microstrip antenna with an orientation of 30° or 45° direction exhibits a slightly higher resonance frequency than that with the 0° orientation before stretching, likely due to the different current distributions in the meshed patch. Although the horseshoe unit cells unravel upon stretching, they unfold differently in the square lattice structure with different orientations. The horseshoe unit cells simultaneously rotate to align and unravel along the stretching (i.e., feeding) direction in the stretchable antenna with the 30° and 45° orientation, whereas the ones in the stretchable antenna with 0° orientation only exhibit unraveling (Figs. 2c and S1a). The predictions from the design by the finite element analysis (FEA) simulation are also verified by the experimental observations. Owing to the additional rotation, the stretchable antenna with the 30° or 45° orientation shows a smaller maximal strain of 2.3% or 2.1% in the horseshoe units of the patch than that of 3.0% in the 0° direction for stretching of 15%. As the maximum strain is below the fracture strain of Cu, the antennas can be stretched further.

Next, the coupled mechanical-electromagnetic properties of the stretchable microstrip antenna are measured to explore their potential for wireless sensing. The reflection coefficient (i.e., the *S*_11_ value) of the antenna is related to the input impedance (*Z*) as $S_{11} = 20\log_{10} \left| {\left( {Z - Z_{0} } \right)/\left( {Z + Z_{0} } \right)} \right|$, where *Z*_0_ is the port impedance of 50 Ω. The input impedance of microstrip antennas is a function of feeding location, which is approximately expressed as $Z = \cos^{2} \left( {\pi x/L} \right)Z\left( {x = 0} \right)$, where L is the length of antennas along the feeding direction, *x* is the inset length, and $Z\left( {x = 0} \right)$ is the input impedance of antennas with feeding on the edge. It should be noted that $Z\left( {x = 0} \right)$ depends on the dimension of antennas and is usually larger than 50 Ω. As the input impedance decreases as the inset length *x* increases, an optimal inset length can be obtained to match the 50 Ω port. Even though the impedance matching is optimized for the solid microstrip antenna, the small *S*_11_ values of the stretchable meshed microstrip antennas imply that a good impedance matching is still achieved. Further improvement on impedance matching is also possible with the optimization of the inset length. The resonance frequency *f* of the patch antenna is inversely proportional to its effective length *L*_eff_ [45] as:

1 $$f = \frac{c}{{2L_{{{\text{eff}}}} \sqrt {\varepsilon_{{{\text{eff}}}} } }},$$

where *c* is the speed of light and *ε*_eff_ is the effective dielectric constant. The effective length is related to the dimension of the patch along the feeding direction with an additional fringing effect for solid microstrip antennas. Owing to the change in the current path, the horseshoe unit cells increase the effective length. The unfolding of the horseshoe unit cells leads to increased effective length and decreased resonance frequency for all three orientations (Figs. 2c and S1). Because of the increased contribution from rotation, the stretchable antenna with the orientation of 30° or 45° exhibits a more linear change in the resonance frequency than the one with the 0° orientation. Compared to the *R*^2^ value of 0.8996 in the linear fitting for the stretchable antenna with the orientation of 0°, the ones with the orientation of 30° (or 45°) showcase a value of 0.999 (or 0.991). The demonstrated linearity in the stretchable antennas is significantly higher than the previous literature reports [32], which is highly desirable for wireless strain sensing or on-body detection of human motions due to the simple calibration. The improved linearity is likely attributed to the additional rotation in the lattice structure during the unraveling process. As shown in Movie S1, the stretchable antenna measures the average mechanical stretching in the antenna from the resonance shift, demonstrating its potential as a wireless strain sensor with the shift to be obtained from remote interrogation. It is also noted that a more uniform deformation to ensure the linearity in the strain sensing can be achieved by a selective bonding only at two ends and/or the use of smaller antennas with higher working frequencies. Compared to the resistive or capacitive strain sensors, the antenna-based wireless strain sensor is of high interest in remoting sensing without wired connections or a power supply. Though wireless strain sensing based on inductive coils has been demonstrated [46], its limited working distance of 2–3 cm can be largely extended to meters with the antenna-based strain sensors. Similar to the previous report [47], the mesh structure slightly decreases the bandwidth of the microstrip antenna from 3% in the solid ones to 1.9–2.8% in the ones with three different mesh orientations, featuring a narrow bandwidth for sensitive strain sensing. Despite the promising application in sensing, the stretchable microstrip antenna with a narrow bandwidth and large resonance shift is not suitable for wireless communication or RF energy harvesting in a predetermined frequency band.

### Hierarchically Structured Microstrip Antennas from Mechanical Assembly

**Fig. 3:**
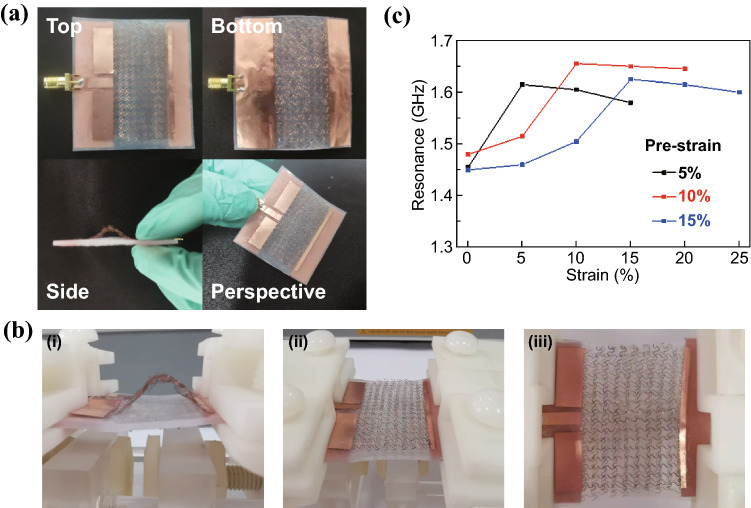
Mechanical-electromagnetic properties of the hierarchically structured microstrip antenna with a single arch generated from different pre-strain levels (5%, 10%, and 15%). **a** Optical images of the hierarchically structured microstrip antenna with a single arch for a pre-strain of 15%. **b** Optical images to show “ordered unraveling” in the hierarchically structured microstrip antenna upon stretching: unraveling of the arch and then the mesh. **c** Measured resonance frequency of the hierarchically structured microstrip antenna with a single arch as a function of the applied tensile strain

To reduce the change in resonance frequency upon stretching, the hierarchically structured stretchable antennas with mechanically assembled 3D structures are exploited (Fig. 3a). Because the mechanically assembled 3D structures largely depend on the level of pre-strain and strategic bonding sites, these two important factors will be investigated in this section. The former includes the study of three pre-strain levels (i.e., 5%, 10%, and 15%), whereas the latter explores selective bonding either at two ends or with an additional center bonding to induce a single- or double-arch structure. As the stretchable antenna with the 0° orientation is associated with a smaller resonance frequency change upon stretching, it is explored in the following studies unless otherwise specified.

The introduction of the 3D pop-up structure further reduces the resonance frequency of the microstrip antenna (Figs. 3b and S2a-c). The air gap between the 3D structure and substrate results in an increased dielectric layer thickness and decreased dielectric constant. According to the transmission line model, the decrease in the dielectric constant leads to an increased resonance frequency, whereas the increase in the dielectric layer thickness results in a decreased resonance frequency through an increased effective length from the fringing effect. However, the influence from the increased thickness on the resonance frequency dominates over that from the reduced dielectric constant to ultimately result in a reduced resonance frequency in the hierarchically structured stretchable antennas. For instance, the resonance frequency is reduced from 1.63 to 1.45 GHz for a pre-strain of 5% before stretching. The arch height increases from 5 to 8.5 mm as the pre-strain increases from 5 to 15% (Fig. S2a), and it slowly decreases with the stretching level at the beginning and then rapidly afterward. For example, for a pre-strain of 15%, the arch height decreases by 1.5 mm upon 5% strain, then by 2.5 mm upon another successive 5% strain, and finally by 4.5 mm with an additional 5% strain. When tensile strain is applied, the hierarchically structured stretchable antenna reveals an “ordered unraveling”, in which the 3D structure arch unravels first to flat and then the horseshoe unfolds (Fig. 2b and Movie S2). The transition between the first and second unraveling occurs when the tensile strain is equal to the pre-strain. The ordered unraveling not only contributes to improved stretchability, but also results in a smaller change in the resonance frequency as it increases in the first unraveling and then decreases in the second (Figs. 3c and S2b-d). In the first unraveling phase, the initial slow increase in the resonance frequency is followed by a rapid increase because of the rapid shape change in the 3D arch (revealed in its measured height, Fig. S2a) near the full unraveling, which is consistent with our prior study [18]. However, the resonance frequency increase in the first unraveling phase is still too large. It is also worth pointing out that the high *S*_11_ values imply a degraded impedance matching, but it can be improved with a dense mesh or by an optimized inset location in future studies. After the patch and ground plane are fully bonded to a pre-strained (10%) substrate, the release of the pre-strain results in the stretchable microstrip antenna with a wavy patch and ground (Fig. S3a). Different from the arched microstrip antenna, the resonance frequency of the stretchable microstrip antenna with a wavy patch and ground decreases monotonously from 1.73 to 1.65 GHz as the tensile strain increases from 0 to 20% due to the flattening of wavy structures and unfolding of serpentine networks (Fig. S3b).

**Fig. 4:**
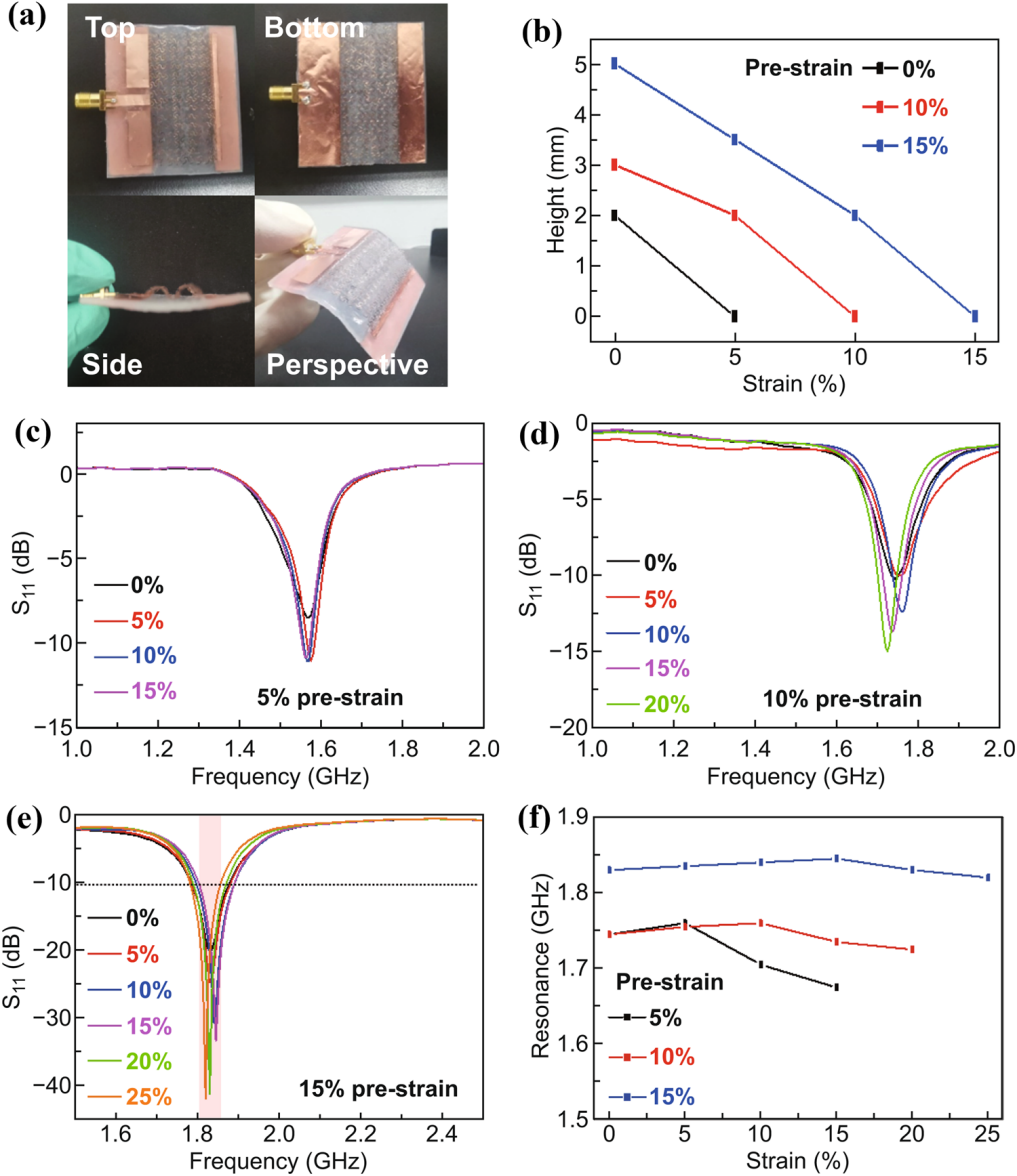
Strain-insensitive hierarchically structured microstrip antennas with a double-arched patch. **a** Optical images of the strain-insensitive hierarchically structured microstrip antenna with a double-arched patch generated from a pre-strain of 15%. **b** Arch height (or half of the amplitude) as a function of the applied tensile strain. **c**–**e** Mechanical-electromagnetic properties of the hierarchically structured microstrip antennas with a double-arched patch for a pre-strain of **c** 5%, **d** 10%, and **e** 15% upon stretching. The operational band with the reflection coefficient (*S*_11_) less than − 10 dB is shaded in pink. **f** Measured resonance frequency of the hierarchically structured microstrip antennas with a double-arched patch upon stretching to highlight the strain-insensitive property in the one from a pre-strain of 15%

With an additional center bonding, a double-arched patch in the hierarchically structured stretchable antenna (Fig. 4a) effectively reduces the change in dielectric constant and dielectric layer thickness for a significantly reduced resonance frequency variation (Fig. 4b–d). It should be noted that the inset length in the hierarchically structured stretchable antenna is optimized to improve impedance matching in case of poor impedance matching. The resonance increase of 0.02 GHz in the double-arched microstrip antenna (Fig. 4e, f) in the first unraveling phase is much smaller than that of 0.12 GHz in its single-arched counterpart (Figs. 3c and S2d) for a pre-strain of 15%. Upon further stretching, the resonance frequency recovers to its initial value to provide an almost unchanged resonance frequency (i.e., < 2% change) over the tensile strain range of 25%. The almost unchanged *S*_11_ curve upon stretching contributes to an operational band of 0.1 GHz (shadow region in Fig. 4e). Although a similar trend is also observed for a pre-strain of 5% or 10%, the resonance frequency variation is much larger than that of 15%. Compared to the arch height of 8.5 mm in the single-arched design for a pre-strain of 15%, the reduced arch height of 5 mm in the double-arched structure helps to improve the stability of the hierarchically structured microstrip antennas upon external perturbations. The smaller air gap in the double-arched microstrip antenna also exhibits improved impedance matching over its single-arched counterpart. Further reduction in the arch height and enhanced impedance matching can be achieved with more selective bonding sites to induce more arches.

In addition to stretching, the double-arched microstrip antenna with a pre-strain of 15% also showcases stable electromagnetic properties (e.g., resonance frequency and radiation patterns) upon bending deformations. With a custom-built bending testing setup (Fig. S4a), the resonance frequency only shows a negligibly small change of 0.012 GHz (0.7%) as the double-arched microstrip antenna is bent over a radius of 14.32 mm (Fig. S4b, c). After 500 bending cycles applied to the double-arched microstrip antenna, no obvious changes are observed in the structure (Fig. S5a) or *S*_11_ curves (Fig. S5b), indicating the good mechanical robustness of the stretchable antenna. Compared to the ungrounded antennas (e.g., monopole, dipole, or loop antenna), the stretchable microstrip antenna with a ground plane exhibits excellent on-body performance. Attaching the double-arched microstrip antennas to different parts of the human body measures its on-body performance (Fig. S6a). A small resonance frequency difference of less than 0.04 GHz is observed between the on-body and off-body measurements (Fig. S6b), indicating the effectiveness of the meshed ground.

### Wireless Communication Performance of the Double-arched Microstrip Antennas

**Fig. 5:**
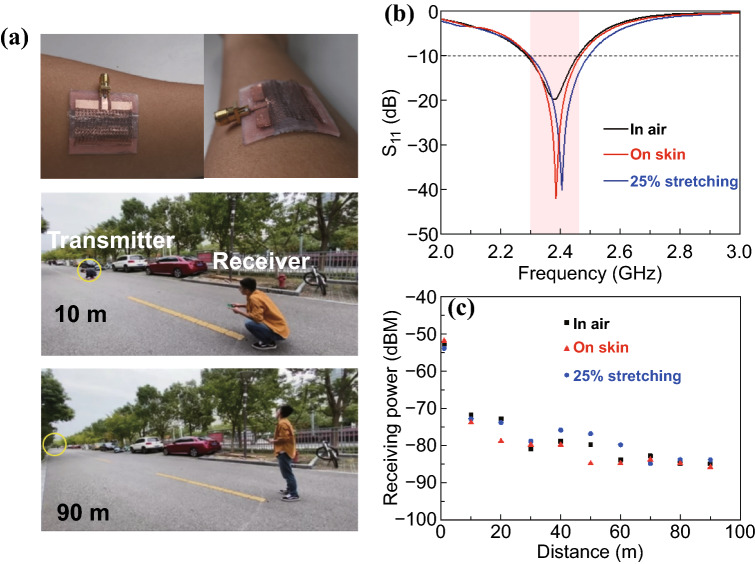
Wireless communication performance of the strain-insensitive hierarchically structured microstrip antenna resonating at 2.4 GHz. **a** Optical images of the strain-insensitive hierarchically structured microstrip antenna conformally attached to the human arm and the experimental setup to evaluate its wireless communication performance. **b** Measured *S*_11_ curves of the strain-insensitive hierarchically structured microstrip antenna in free-space or on the human arm with/without a tensile strain of 25%. **c** Measured receiving power by the strain-insensitive stretchable antenna from a transmitter positioned at different distances

The strain-insensitive electromagnetic property of the stretchable microstrip antennas in the intrinsically narrow bandwidth is highly desirable for stable wireless communication and effective RF energy harvesting, especially for on-body applications. Because 2.40–2.48 GHz is a widely used frequency range in wireless communication (e.g., Bluetooth and Wi-fi), the stretchable antennas with a stable resonance frequency around 2.45 GHz can be directly leveraged for wireless data transmission or powering. The double-arched microstrip antenna with a pre-strain of 15% can be easily attached to the curvilinear surface of human arms without causing discomfort (Fig. 5a). Improved adhesion between the antenna and arm can be achieved by coating a thin adhesive Silbione layer on the wavy ground. Because of the reduced antenna dimension from 43.9 × 35.5 to 31.9 × 25.5 mm^2^ along with the inset length optimization, the double-arched microstrip antenna resonates at 2.45 GHz, as confirmed by the experimental measurements (Fig. 5b). The negligibly small influence of human bodies on the resonance frequency of the antenna also demonstrates the effectiveness of the meshed ground in this new design. Further improvement in the on-body performance (e.g., screening effect and radiation directionality) can be achieved with a dense ground mesh.

The wireless communication performance of the double-arched microstrip antenna is measured with a commercial RF evaluation kit consisting of a transmitter and a receiver (SmartRF06) (Fig. S7). The transmitter integrated with a PCB-based omnidirectional antenna and a CC2538 RF chip is programmed to transmit RF power at − 3 dBm (0.50 mW). The receiver with a sensitivity of − 100 dBm is integrated with the double-arched microstrip antenna. The stretchable microstrip antenna was either placed in the air or on the human skin and upon mechanical stretching. The communication performance is evaluated in the open space at a university campus (Fig. 5a). Although the receiving power decreases rapidly with the communication distance for both the in-air and on-skin case, the receiver is still able to receive − 100 dBm at a distance of ~ 100 m (Fig. 5c). It is believed that the working distance can be improved further by increasing the transmitting power of the source. The stretchable microstrip antenna in this work exhibits improved communication performance in the free space over the previous demonstrations (e.g., with a stretchable monopole antenna [40]). Compared to the received power of − 75 dBm by the monopolar antenna from a 1 dBm transmitter at a distance of 20 m, our stretchable microstrip antennas demonstrate a higher received power of − 72 dBm from a transmitter with an even lower power of − 3 dBm. Moreover, the significantly enhanced on-body performance of the stretchable microstrip patch antenna further results in a small difference in the receiving power between the on-body and free-space demonstrations. For example, the receiving power difference at a distance of 20 m between the “in-air” and “on-body” case is ~ 5 dBm, much lower than that for the monopole antenna (~ 12 dBm). These improved on-body performance parameters in wireless communication are attributed to the almost unchanged resonance frequency and radiation properties of the stretchable microstrip antenna (Fig. 5b). The wireless communication performance of the stretchable microstrip antenna upon deformations was also investigated. Mechanical stretching leads to a slight change of the receiving power, which can be explained by negligible resonance frequency change of the stretchable microstrip antenna upon stretching.

## Conclusions

In summary, we have introduced a hierarchically structured stretchable microstrip antenna with horseshoe unit cells arranged in a square lattice structure that is further mechanically assembled into a 3D layout. The resulting stretchable antenna showcases tunable, especially strain-insensitive mechanical-electromagnetic properties with improved overall stretchability. In particular, an almost unchanged resonance frequency with a shift of < 0.02 GHz is demonstrated within the tensile strain range from 0 to 25%. To the best of our knowledge, this is the first demonstrated stretchable microstrip antenna that has almost unchanged resonance frequency over a tensile strain range of over 25%. The stretchable microstrip patch antennas with the strain-insensitive resonance frequency and enhanced stretchability extend their application from wireless sensing to stable on-body wireless communication and effective RF energy harvesting. Additionally, the design approach based on the coupled mechanical-electromagnetic simulations also allows us to identify the stretchable microstrip antennas as wireless sensors with enhanced linearity. This work provides a powerful toolkit with coupled mechanical-electromagnetic simulations and cost-effective manufacturing approaches to design stretchable microwave components/devices for integrated stretchable systems.

## Supplementary Information

Below is the link to the Supplementary Information.

Supplementary Information 1 (MP4 488 kb)

Supplementary Information 2 (MP4 971 kb)

Supplementary Information 3 (PDF 795 kb)
