# Supplementary material for: Strain-Insensitive Hierarchically Structured Stretchable Microstrip Antennas for Robust Wireless Communication
Source: Nanomicro Lett. 2021 Apr 9;13:108. doi: 10.1007/s40820-021-00631-5 (PMC8035380; doi:10.1007/s40820-021-00631-5)
Supplement: Supplementary file 3 — Supplementary Information 3 (PDF 795 kb) [file 40820_2021_631_MOESM3_ESM.pdf]

Supporting Information for

Strain-Insensitive Hierarchically Structured Stretchable Microstrip Antennas for Robust Wireless Communication

Jia Zhu[1, *], Senhao Zhang[1, 2, 3], Ning Yi[4], Chaoyun Song[5], Donghai Qiu[3], Zhihui Hu[1], Bowen Li[1], Chenghao Xing[6], Hongbo Yang[2, 3], Qing Wang[4], Huanyu Cheng[1, 4, *]

[1]Department of Engineering Science and Mechanics, The Pennsylvania State University, University Park, Pennsylvania 16802, USA

[2]School of Biomedical Engineering (Suzhou), Division of Life Sciences and Medicine, University of Science and Technology of China, Hefei, 230022, P. R. China

[3]Suzhou Institute of Biomedical Engineering and Technology, Chinese Academy of Science, Suzhou, 215011, P. R. China

[4]Department of Materials Science and Engineering, The Pennsylvania State University, University Park, Pennsylvania 16802, USA

[5]School of Engineering and Physical Sciences, Heriot-Watt University, University Park, Edinburgh EH14 4AS, Scotland, UK, United Kingdom

[6]Department of Electrical Engineering, The Pennsylvania State University, University Park, Pennsylvania 16802, USA

Jia Zhu and Senhao Zhang contributed equally to this study and share the first authorship

*Corresponding authors. E-mail: jmz5364@psu.edu (J. Z.), Huanyu.Cheng@psu.edu (H. C.)

S1 Transmission Line Model for Microstrip Antennas

The resonance frequency f of the microstrip patch antenna with a width and length of w and L in the patch can be designed as a function of the effective dielectric constant ε_{eff} and the effective length L_{eff} as in Eq. 1. The effective dielectric constant ε_{eff} and the effective length L_{eff} can be obtained from

$$\varepsilon_{eff} = \frac{\varepsilon_r+1}{2} + \frac{\varepsilon_r-1}{2}\left(1+12\left(\frac{h}{w}\right)\right)^{-\frac{1}{2}}, \qquad \text{(S1)}$$

$$\Delta L = 0.412h\frac{(\varepsilon_{eff}+0.3)(\frac{w}{h}+0.264)}{(\varepsilon_{eff}-0.258)(\frac{w}{h}+0.8)}, \qquad \text{(S2)}$$

$$L_{eff} = L + 2\Delta L, \qquad \text{(S3)}$$

where ε_r and h are the relative dielectric constant and dielectric layer thickness, respectively. The effective length L_{eff} is large than the physical length L of microstrip patch antennas due to the fringing effect.

S2 Supplementary Figures

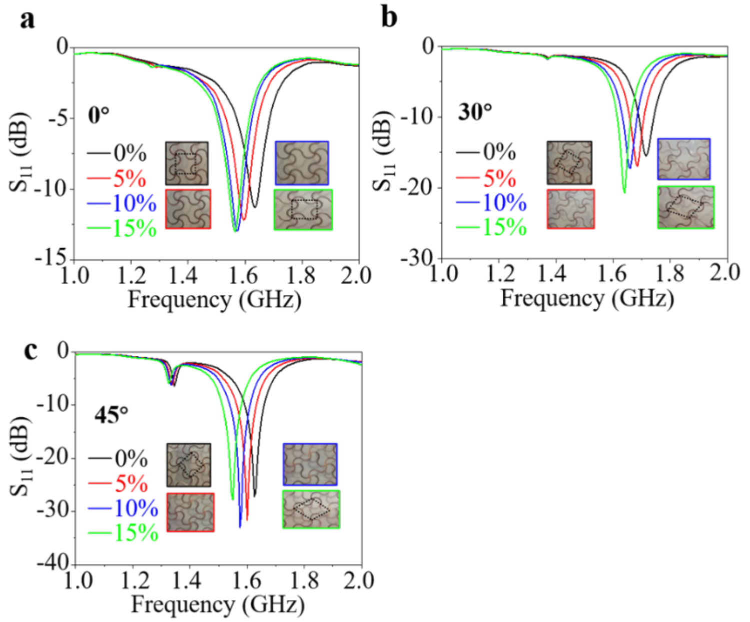

Fig. S1 Measured S_{11} curves of the meshed microstrip antenna with different orientations (0˚, 30˚, and 45˚) upon stretching. The unit cell of the mesh is orientated along the **(a)** 0˚, **(b)** 30˚, and **(c)** 45˚. The optical images in the inset show the deformed meshed structure upon stretching.

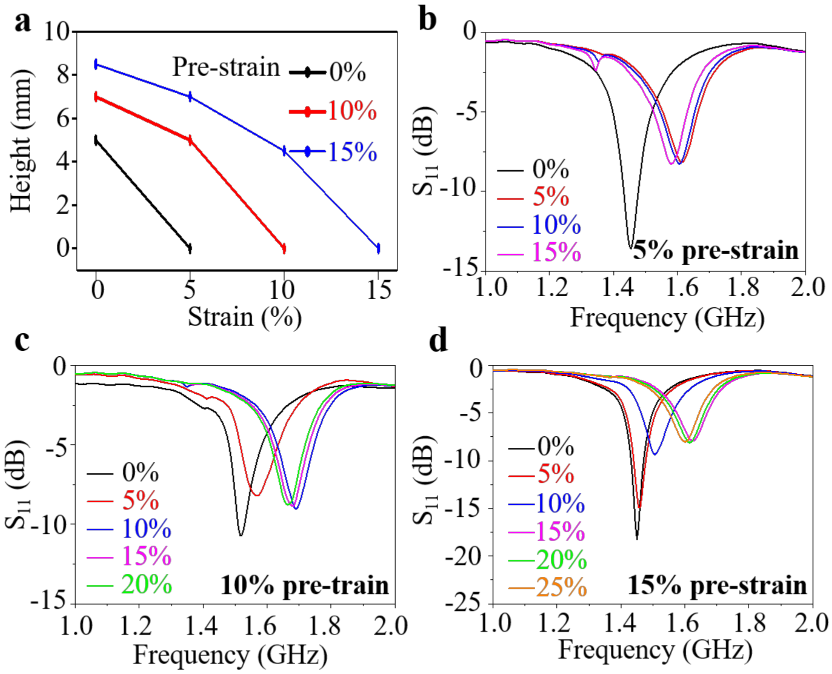

Fig. S2 Mechanical-electromagnetic properties of the hierarchically structured microstrip antenna with a single arch. **a** Height of the arched patch as a function of the applied tensile strain. **b-d.** Measured S_{11} curves of the hierarchically structured microstrip antenna with a single arch for a pre-strain of **b** 5%, **c** 10%, and **d** 15% upon stretching

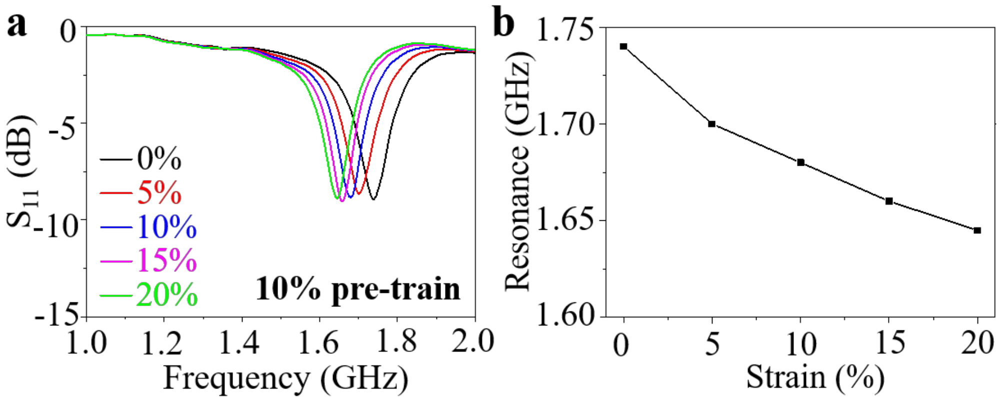

Fig. S3 Mechanical-electromagnetic properties of stretchable microstrip antennas with a wavy patch and ground (pre-strain of 10%)

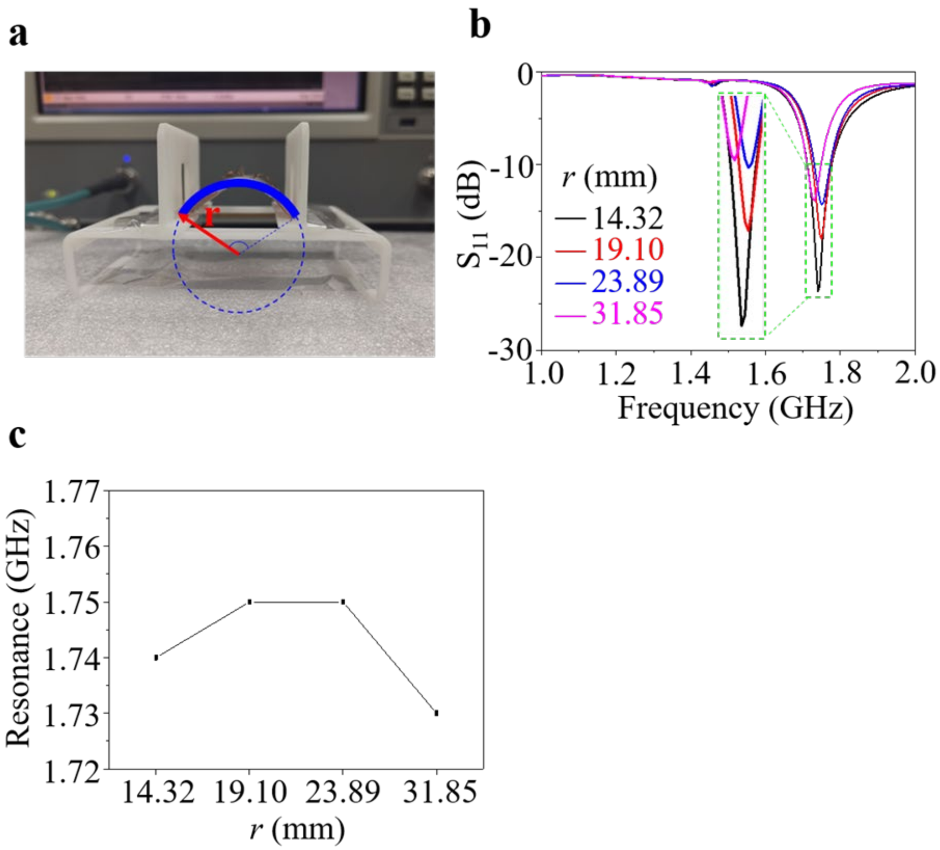

Fig. S4 Bending performance of the hierarchically structured microstrip antenna with a double-arched patch for a pre-strain of 15%. **a** Experimental setup for the bending test. **b** Measured S_{11} curves of the hierarchically structured microstrip antenna with a double-arched patch upon bending deformation for different radii. **c.** Measured resonance frequency as a function of the bending radius from the hierarchically structured microstrip antenna with a double-arched patch

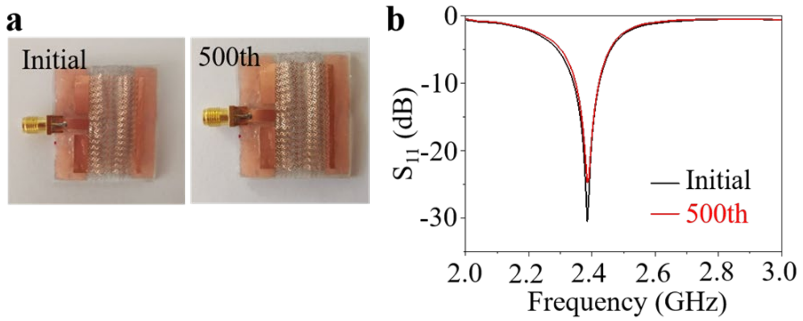

Fig. S5 (**a**) Optical images and (**b**) S_{11} curves of hierarchically structured microstrip antennas with a double-arched patch before and after 500 bending cycles

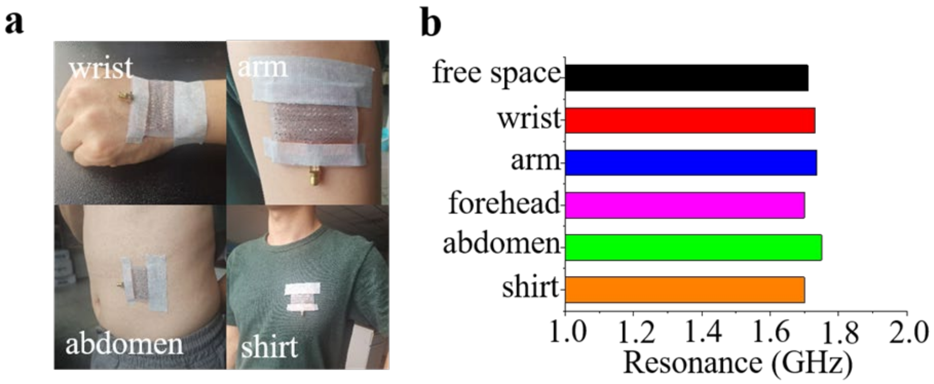

Fig. S6 On-body electromagnetic properties of the hierarchically structured microstrip antenna with a double-arched patch. **a.** Optical images of the hierarchically structured microstrip antenna with a double-arched patch attached to different parts of human bodies, including the wrist, arm, abdomen, and shirt over the chest. **b.** Measured resonance frequency of the hierarchically structured microstrip antenna with a double-arched patch placed at different locations

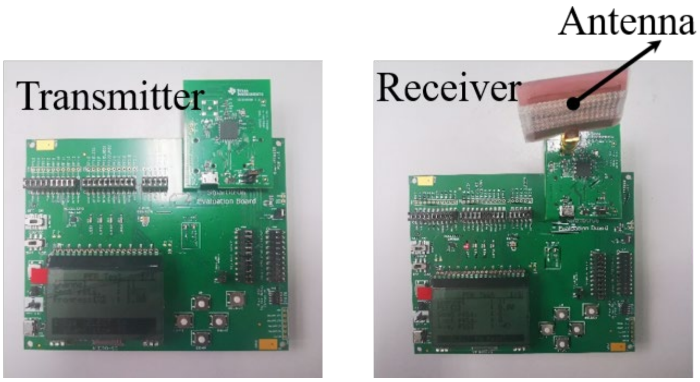

Fig. S7 RF transmitter and receiver used to evaluate the wireless communication performance of the hierarchically structured microstrip antenna.

Movie S1 (separate file). Demonstration of the strain sensing capability from the meshed microstrip antenna.

Movie S2 (separate file). "Ordered unraveling" in the hierarchically structured microstrip antenna with a single-arched patch upon stretching.
